# Frequency of Anti-SARS-CoV-2 Antibodies in Various Occupational Sectors in an Industrialized Area of Northern Italy from May to October 2020 †

**DOI:** 10.3390/ijerph18157948

**Published:** 2021-07-27

**Authors:** Alberto Modenese, Tommaso Mazzoli, Nausicaa Berselli, Davide Ferrari, Annalisa Bargellini, Paola Borella, Tommaso Filippini, Isabella Marchesi, Stefania Paduano, Marco Vinceti, Fabriziomaria Gobba

**Affiliations:** 1 Department of Biomedical, Metabolic and Neural Sciences, University of Modena and Reggio Emilia, 41125 Modena, Italy; alberto.modenese@unimore.it (A.M.); nausicaa.berselli@unimore.it (N.B.); annalisa.bargellini@unimore.it (A.B.); paola.borella@unimore.it (P.B.); tommaso.filippini@unimore.it (T.F.); isabella.marchesi@unimore.it (I.M.); stefania.paduano@unimore.it (S.P.); marco.vinceti@unimore.it (M.V.); 2 Department of Public Health, National Health Service, 41126 Modena, Italy; tommymazzoli@hotmail.it (T.M.); da.ferrari@ausl.mo.it (D.F.)

**Keywords:** SARS-CoV2, COVID-19, SARS-CoV2 antibody, occupational exposure, occupational health, work-related risk

## Abstract

The results of a voluntary screening campaign for the presence of anti-SARS-CoV-2 serum antibodies are presented, performed on workers in the highly industrialized province of Modena in northern Italy in the period 18 May–5 October 2020. The employment activities of the subjects that tested positive for anti-SARS-CoV-2 IgM and/or IgG antibodies were determined and classified using the International Standard Industrial Classification of All Economic Activities (ISIC). The distribution across different sectors was compared to the proportion of workers employed in the same sectors in the province of Modena as a whole. Workers with anti-SARS-CoV-2 serum antibodies were mainly employed in manufacturing (60%), trade (12%), transportation (9%), scientific and technical activities (5%), and arts, entertainment and recreation activities (4.5%). Within the manufacturing sector, a cluster of workers with positive serological tests was observed in the meat processing sector, confirming recent data showing a possible increased risk of SARS-CoV-2 infection in these workers.

## 1. Introduction

During the whole SARS-CoV-2 pandemic, but especially during the first wave, the highly industrialized regions of northern Italy were the most severely affected areas in Italy, while these differences were less marked during the second wave of the pandemic, roughly from Autumn 2020. In Emilia-Romagna, one of these regions, a cumulative number of 352,902 SARS-CoV-2 infections was confirmed on 11 April 2021, representing 9.3% of the total 3,772,617 cases recorded in Italy since the beginning of the epidemic [1].

During the initial phases of the pandemic in Italy (March–May 2020), the Italian Government imposed strict confinement measures and a temporary suspension of all non-essential activities, including manufacturing and services, as well as face-to-face lessons in schools and universities (the “lockdown”). Subsequently, from the second half of May, there was a progressive relaxation of these restrictions for a large number of manufacturing activities, and many workers returned to work [2]. However, workplaces were identified as sites with a high risk of infection right from the start. Outbreaks of the virus were observed among health workers but also in meat and poultry processing facilities and in other sectors, such as tourism, retail and hospitality, transport, and construction, raising the issue of the need for additional preventive measures to reduce/avoid the risk SARS-CoV-2 infection in the workplace [3].

Among the preventive measures applied, one of the most important was the prompt identification of SARS-CoV-2 infections occurring in the workplace, allowing further actions including contact tracing. Due to the insufficient availability of rhino-pharyngeal swab testing for molecular SARS-CoV-2 infection diagnosis through Reverse Transcriptase–Polymerase Chain Reaction (RT-PCR), a screening campaign was proposed involving anti-SARS-CoV-2 antibody testing in workers followed by a diagnostic swab in case of a positive result [4]. This screening has been extensively applied to healthcare workers (HCWs) since the beginning of the epidemic, highlighting a high proportion of infections in this group. Currently HCWs account for more than 3.5% of the total number of registered cases in Italy [1], but at the beginning of the COVID-19 pandemic, this figure was as high as 12% and even higher in some cases [5]. Unfortunately, for practical reasons, similar extensive systematic screening was not possible in other employment sectors, but a voluntary screening campaign was promoted for workers. Several employers joined the campaign, offering their employees the chance to participate. 

The aim of this article is to present an overview of the results of this campaign, and to describe the frequency of positive anti-SARS-CoV-2 antibody tests in non-healthcare workers employed in workplaces in the province of Modena [6] as part of the serological screening campaign undertaken in private sector companies between May and October 2020. In this period, new COVID-19 cases increased by about 100,000 units in Italy [7,8], of which around 1% occurred in the province of Modena [9].

## 2. Materials and Methods

As described above, in the early phases of the COVID-19 pandemic in Italy, due to the inadequate availability of rhino-pharyngeal swab testing for molecular diagnosis of COVID-19, a screening campaign of SARS-CoV-2 infection in workers was promoted through anti-SARS-CoV-2 antibody testing, and several companies voluntarily took part. According to a local law in force in Emilia-Romagna [4], all positive cases (either IgM or IgG) identified in workers other than healthcare personnel (managed separately) had to be notified to the Occupational Health and Safety Section (OHSS) of the local Department of Public Health (DPH). Subsequently these workers underwent a rhino-pharyngeal swab for molecular COVID-19 diagnosis through RT-PCR. The notification of cases of antibody positivity to the OHSS was the responsibility of the authorized laboratories performing the tests, with the involvement of the general practitioners (GPs) or of the Occupational Physicians (OPs) of the companies. We collected all the notifications received by the OHSS of the Modena DPH in the period 18 May–5 October 2020 relating to workers employed in companies within the province of Modena. For those cases notified by labs and referred to GPs, only the main socio-demographic data of the subjects was available, while for positive tests notified by labs involving OPs as part of screening performed within companies, occupation-related information was also available.

The activities performed by workers included in the study were classified using the International Standard Industrial Classification of All Economic Activities (ISIC) [10].

Some occupational categories were not included in this analysis, which focuses primarily on the work activities performed in private companies within the province. The main reasons for these exclusions are as follows:

- HCWs, ISIC code “Q. Human health and social work activities”, were not considered, as these are managed separately and not included in the database of this screening campaign.
- ISIC code “O”, i.e., Public administration and defense activities—this sector includes police and military personnel, who were not considered, since, like HCWs, they are managed separately and were not included in this screening campaign, and Public Administration personnel. In the latter case, it should be noted that almost all were allowed to work from home during the strict lockdown period in Italy (March–June) and, in any case, direct contact with the general public, or other workers, was formally forbidden.
- ISIC code “P”, i.e., Education—as in the case of Public Administration personnel, no contact, or very limited direct contact, was made by these workers with students or other personnel, as schools remained closed in Italy during the whole period of March–June 2020, and during the summer break.
- ISIC code “T”, i.e., Activities of households as employers, undifferentiated goods- and services-producing activities of households for own use. This category includes mainly domestic services workers, frequently migrant workers employed by households, with a limited possibility of being involved in the screening campaign.

Considering these exclusions, the source population included all workers in the province of Modena employed in companies classified with codes A to N, plus the codes R, S and U, according to the ISIC classification. We estimated the total number of active workers in these sectors in the province of Modena based on the publicly available data of the National Institute for Insurance against Accidents at Work (INAIL) [11] for industrial, commercial and services activities, and of the Chamber of Commerce [12] for data on agricultural workers. The percentage of the workforce active in the province of Modena according to the different ISIC codes was then calculated. Finally, we attributed the ISIC codes to those workers with a positive result, i.e., IgG+ and/or IgM+, for the serological test for the detection of anti-SARS-CoV-2 antibodies. For manufacturing activities (section C of the ISIC classification) i.e., the most common employment activities in the province of Modena [13], we expanded the classification according to the different ISIC Section C sub-codes. 

## 3. Results

**Figure 1:**
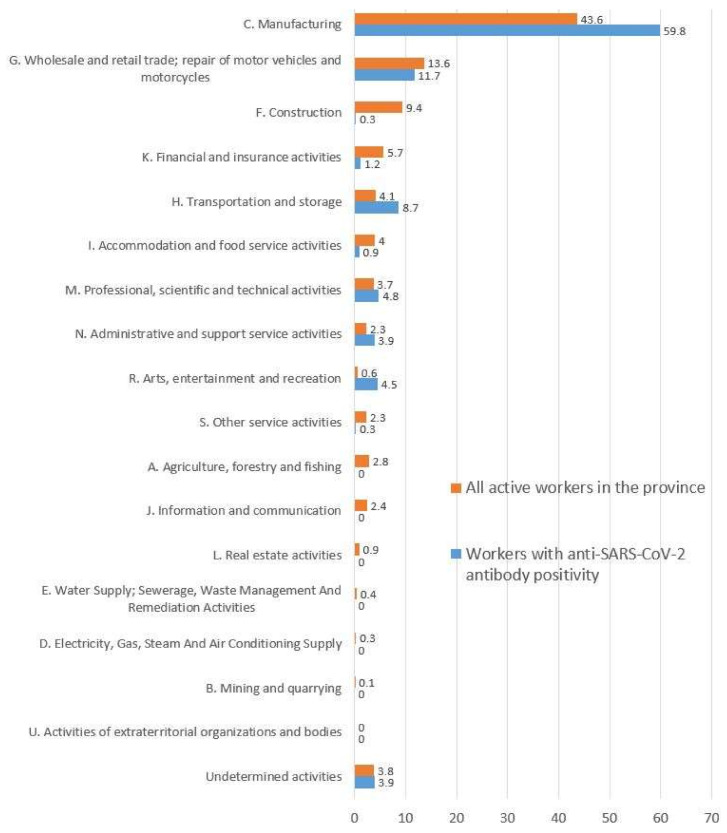
Percentage distribution of workers employed in the province of Modena according to ISIC sector: all active workers compared to workers with a positive anti-SARS-CoV-2 serological antibody test result.

Based on INAIL and Chamber of Commerce data, after the exclusion of the ISIC codes O, P, Q and T for the abovementioned reasons, we calculated 246,608 active workers in the province of Modena. The percentage distribution of these workers across the different employment sectors classified according to the ISIC codes are presented in Figure 1 (in orange).

Of these subjects, 0.45% (n = 1103) gave a positive anti-SARS-CoV-2 IgG and/or IgM result based on the results of the voluntary serological test campaign conducted in the period 18 May–5 October 2020. Of these cases, 34.6% tested positive for both IgG and IgM, 50.6% for IgG only, and the 14.8% for IgM only.

All workers that tested positive underwent a RT-PCR rhino-pharyngeal swab: in 34 of the cases, i.e., 3.1%, the test result was positive, and a diagnosis of SARS-CoV-2 infection was made.

As described in the Materials and Methods section, the reporting of the cases of antibody positivity to the OHSS of the local DPH was the responsibility of the authorized labs performing the tests, involving either the GPs or the OPs of the companies. For 732 subjects, antibody positivity was reported by the labs with the involvement of GPs, while the remaining 371 cases involved the OPs of the companies taking part in the screening campaign. As already discussed, the ISIC classification of the companies was available only for these 371 workers; however, due to technical problems, the information was incomplete for a further 48 workers. Consequently, a total sample of 333 subjects was available for further processing to evaluate the differences in the distribution of workers according to ISIC code compared to the distribution in the province. Figure 1 shows the distribution of workers with a positive anti-SARS-CoV-2 antibody test result according to the different ISIC codes of their companies (in blue) (Figure 1). 

About 60% were employed in manufacturing activities (ISIC code = C), 12% worked in wholesale and retail trade (ISIC code = G), 9% worked in transportation and storage (ISIC code = H), 5% in professional, scientific and technical activities (ISIC code = M), and 4.5% in arts, entertainment and recreation activities (ISIC code = R). A total of 10.5% were employed in all remaining work activities, distributed across the ISIC codes F, I, K, N, including 13 workers for whom it was not possible to determine the ISIC code. No positive cases were reported in the sectors corresponding to the ISIC codes A, B, D, E, J, L and U. For sectors corresponding to the ISIC codes C “manufacturing”, H “transportation and storage”, M “professional, scientific and technical activities”, N “administrative and support services activities”, and R “arts, entertainment and recreation”, the percentage of workers with a positive anti-SARS-CoV-2 serological antibody test result was higher than the overall percentage distribution of active workers in the province of Modena. On the contrary, for the ISIC activities A “agriculture, forestry and fishing”, F “construction”, G “wholesale and retail trade (including repair of motor vehicles)”, J “accommodation and food service activities”, and K “financial and insurance activities”, the proportion of “positive” workers was clearly lower than the current distribution of the workforce in the province. For the remaining ISIC codes, no clear conclusion can be drawn as the proportion of workers employed in these sectors in the province is very low, and the number of positive cases is very small, when not zero (Figure 1).

Manufacturing activities with the general ISIC code “C” were examined in more detail according to the ISIC sub-codes. Table 1 shows the percentage of manufacturing workers that tested positive for anti-SARS-Cov-2 antibodies classified according to the specific ISIC sub-codes, and the distribution of all workers engaged in these activities in the province of Modena. Only sub-codes for which at least one positive case was reported are included in Table 1. 

For activities corresponding to the ISIC sub-codes C.10 “Manufacture of food products”, C.26 “Manufacture of computer, electronic and optical products”, C.28 “Manufacture of machinery and equipment not elsewhere classified”, C.29 “Manufacture of motor vehicles, trailers and semi-trailers”, and C.33 “Repair and installation of machinery and equipment”, the percentage of workers with a positive serology result was higher than the overall percentage distribution of active workers in the province of Modena (Table 1). On the other hand, for the ISIC sub-codes C.14 “Manufacture of wearing apparel”, C.20 “Manufacture of chemicals and chemical products”, C.23 “Manufacture of other non-metallic mineral products”, and C.25 “Manufacture of fabricated metal products, except machinery and equipment”, the percentage of “positive” workers was largely lower than the current distribution of the manufacturing workforce in the province. For the remaining ISIC sub-codes of the manufacturing sector, no conclusions can be drawn as the proportion of workers employed in these specific activities in the province is very low, and the number of positive cases insufficient (Table 1).

## 4. Discussion

According to the results of the described voluntary screening campaign performed in the highly industrialized province of Modena in northern Italy, during the latter part of the first COVID-19 wave (second half of May–first half of October 2020), 1103 subjects tested positive for anti-SARS-CoV-2 antibodies (either IgG and/or IgM). The percentage of the overall population of active workers is 0.45%. This percentage seems lower than expected; for a more adequate comparison it should be observed that in the almost five months considered, 1110 cases of SARS-CoV-2 infections were reported in the whole province of Modena, including young, elderly and non-working people [7,8,9]. As a consequence, taking into account that only working subjects were considered, the 1103 cases of serological antibody positivity seem largely in line with the period of lower infection rates observed (compared to the extremely high infection rates during the first and second waves) [1,14]. Furthermore, as stated in the Materials and Methods section, we also excluded some specific occupational categories at increased risk of SARS-CoV-2 infection [3,14], such as military and police personnel, educational workers, domestic services personnel, and HCWs. These latter groups of workers significantly contributed to the overall numbers of COVID-19 cases, in particular during the first wave of the pandemic, with a prevalence of infection estimated at 12% [14]. As a result, the serological antibody positivity rate is expected to be high; recent Italian data collected in the same period as our study showed a 90-day IgM positivity rate of 11.5% and 2.4% for IgG positivity [15]. In our sample of non-healthcare workers, we found that 50% tested positive for IgM (alone or together with IgG); this may indicate that half of these workers had a relatively recent SARS-CoV-2 infection [16].

Another important reference for the interpretation of our data is the report of the Italian National Institute of Statistics, indicating a prevalence of anti-SARS-CoV-2 serological antibodies (considering IgG only) of 2.8%, with no difference between employed and unemployed subjects, registered in a period of less than two months between the end of May and the first half of July 2020 [17]. These data clearly indicate that our serological screening campaign reached only a fraction of the total number of workers employed in the province. However, it should be considered that during the period March–June 2020, many of the employment sectors we analyzed experienced severe restrictions of their activities due to the national lockdown. In addition, after June, many office workers did not return to their workplaces but continued working from home [14]. 

Despite this limitation, our study highlights some important relationships between occupation and the risk of SARS-CoV-2 infection. To date, data on the diffusion of the new coronavirus and on the specific risk factors in work activities other than health care have been scarce and limited in scope. In principle, all workers involved in providing services to the general public can be considered potentially at risk from infection [18]. Moreover, the risk can be considered higher for those workers involved in activities with close physical proximity, especially in indoor settings or with shared transport or accommodation [3,14,18]. Recent Italian data from INAIL indicates that, among all the applications submitted for COVID-19 compensation, about thirty thousand in total at the time of the study, 71.6% came from Human health and social work activities, i.e., the sector including HCWs, 10.4% from Public administration and defense activities, 4.1% from Administrative and support service activities, 2.6% from the Manufacturing sector, 2.4% from Accommodation and food service activities, 2.1% from the Agriculture, forestry and fishing sector, and only 6.8% from all the other employment activities [14].

In our previous seroprevalence analysis, conducted over a larger geographical area based on data obtained from a single lab, we found an overall prevalence of anti-SARS-CoV-2 positivity of 4.7%, which was higher in women (5.4%) and in the oldest age groups (7.3% between 60 and 69, and 11.8% in those ≥70). Among the occupational categories, we found the highest seroprevalence in HCWs (8.8%), vehicle dealers and repairers (5.2%), and sports sector workers (4.0%) [19].

The context of our research can be considered very much suited to the study of the role of occupation, and in particular industrial-related occupations, in the spread of SARS-CoV-2 in Italy. The province of Modena is one of the most industrialized areas of Italy, with a significantly higher proportion of workers engaged in industrial activities compared to the average in Italy (40% compared to a national percentage of 30%) [13], and is also located in the part of the country most significantly impacted by the COVID-19 outbreak, especially during the first wave of the pandemic. Furthermore, it should also be considered that this province is densely populated and polluted, factors potentially playing a role in the spread of SARS-CoV-2 [20,21]. Our study highlights a higher rate of anti-SARS-COV-2 antibody positivity in the manufacturing and transportation sectors, as well as in professional, scientific and technical activities (ISIC code M), administrative and support services activities (ISIC code N), and in recreation (including arts and sports) activities (ISIC code R) when compared to the current distribution of workers in the same employment activities in the province. The data related to arts and recreation activities, including sports, is particularly interesting. We found an anti-SARS-CoV-2 positivity rate about eight times higher than the proportion of workers employed in this sector in the province as a whole, even if the data should be interpreted with caution, considering that the results are based on a voluntarily screening campaign. By expanding the analysis within the manufacturing sector, i.e., the employment group with the highest number of active workers in the province of Modena, and the highest number of “positive” workers, we found that for the ISIC sub-codes related to the production of food, electronic equipment, other machinery (including repair and installation) and of motor vehicles, the percentage of workers employed in these activities with anti-SARS-CoV-2 serological antibody positivity was higher than the overall percentage distribution of manufacturing workers in the province. One of the jobs considered at higher risk for possible SARS-CoV-2 infection is the meat processing sector [22]. Workers employed in activities within the ISIC sub-code C.10 “Manufacture of food products” comprise about 11% of the manufacturing workforce in the province of Modena, and 13% of the cases of anti-SARS-CoV-2 antibody positivity reported to the OHSS of the Modena DPH for the manufacturing sector: 23 of the 25 cases included in the C.10 sector were employees at meat processing factories. As previously highlighted, the interpretation of these results is limited by the fact that workers in the manufacturing sector are the most represented group in our sample of “positive” workers as well as in the source population. Moreover, it cannot be excluded that, on average, these workers have been tested more frequently for anti-SARS-CoV-2 antibodies than workers in other employment groups. This may also have occurred because the manufacturing sector includes a high number of employees who continued going to work even during lockdown and are more likely to have taken part in the screening campaign.

## 5. Conclusions

The analysis of the distribution of anti-SARS-CoV-2 serological antibody positivity among workers other than HCWs in a highly industrialized area of Italy revealed a possible higher risk of infection in the manufacturing sector, and in particular in meat processing activities, and transportation sectors, activities that generally continued during the Italian national lockdown from March to June 2020. Another finding possibly deserving further attention relates to arts and recreation activities, including sports, where we found an anti-SARS-CoV-2 positivity rate about eight times higher than the proportion of workers employed in this sector.

## Figures and Tables

**Table 1:** Comparison between the distribution of workers employed in manufacturing activities in the province of Modena and the 199 workers who tested positive for anti-SARS-CoV-2 antibodies employed in these activities in the same province.

ISIC Category	Workers Employed in the Manufacturing Sector in the Province of Modena: % (Total Number = 107,434)	Workers Who Tested Positive for Anti-Sars-Cov-2 Antibodies: % (n)
C.10 Manufacture of food products	10.9 (11,748)	12.6 (25)
C.14 Manufacture of wearing apparel	5.5 (5939)	1.5 (3)
C.16 Manufacture of wood and of products of wood and cork, except furniture; manufacture of articles of straw and plaiting materials	1.3 (1401)	1.0 (2)
C.17 Manufacture of paper and paper products	1.2 (1260)	1.5 (3)
C.20 Manufacture of chemicals and chemical products	2.6 (2781)	1.0 (2)
C.21 Manufacture of pharmaceuticals, medicinal chemical and botanical products	0.8 (865)	1.0 (2)
C.22 Manufacture of rubber and plastics products	2.1 (2203)	2.0 (4)
C.23 Manufacture of other non-metallic mineral products	14.7 (15,771)	9.5 (19)
C.24 Manufacture of basic metals	0.8 (829)	0.5 (1)
C.25 Manufacture of fabricated metal products, except machinery and equipment	12.4 (13,326)	5.5 (11)
C.26 Manufacture of computer, electronic and optical products	3.0 (3180)	17.6 (35)
C.28 Manufacture of machinery and equipment n.e.c.	23.2 (24,955)	31.7 (63)
C.29 Manufacture of motor vehicles, trailers and semi-trailers	8.6 (9285)	10.0 (20)
C.33 Repair and installation of machinery and equipment	2.8 (2975)	4.5 (9)

## Data Availability

The data presented in this study are available on request from the corresponding author. The data are not publicly available due to privacy restrictions.
